# Emerging technologies in adipose tissue research

**DOI:** 10.1080/21623945.2023.2248673

**Published:** 2023-08-20

**Authors:** Dimiter Avtanski, Nikola Hadzi-Petrushev, Slavica Josifovska, Mitko Mladenov, Varun Reddy

**Affiliations:** a Friedman Diabetes Institute, Lenox Hill Hospital, New York, NY, USA; b Institute of Bioelectronic Medicine, Feinstein Institutes for Medical Research, Manhasset, NY, USA; c Donald and Barbara Zucker School of Medicine at Hofstra/Northwell, Hempstead, NY, USA; d Faculty of Natural Sciences and Mathematics, Institute of Biology, “Ss. Cyril and Methodius” University, Skopje, North Macedonia; e New York Institute of Technology College of Osteopathic Medicine, Old Westbury, NY, USA

**Keywords:** Adipose tissue, research, technologies, analyses, biomarkers

## Abstract

Technologies are transforming the understanding of adipose tissue as a complex and dynamic tissue that plays a critical role in energy homoeostasis and metabolic health. This mini-review provides a brief overview of the potential impact of novel technologies in biomedical research and aims to identify areas where these technologies can make the most significant contribution to adipose tissue research. It discusses the impact of cutting-edge technologies such as single-cell sequencing, multi-omics analyses, spatial transcriptomics, live imaging, 3D tissue engineering, microbiome analysis, *in vivo* imaging, and artificial intelligence/machine learning. As these technologies continue to evolve, we can expect them to play an increasingly important role in advancing our understanding of adipose tissue and improving the treatment of related diseases.

## 1. Introduction

Nowadays, adipose tissue (AT) is recognized as an important endocrine organ, and attention to it is rapidly increasing owing to the worldwide obesity epidemic. AT research is a dynamically evolving field, and new emerging technologies have revolutionized our understanding of this complex tissue. Single-cell sequencing technologies allow simultaneous measurement of multiple modalities at the single-cell level. Multi-omics analyses provide direct measurements of biological phenotypes that uncover scientific insights that cannot be obtained from single omics methods alone. Spatial transcriptomics accurately reveals the spatial organization of cell types in tissues. Live imaging allows us to observe dynamic biological processes in real time and at high resolution. Three-dimensional (3D) tissue engineering techniques help create complex tissue structures that mimic the native environment of cells and tissues *in vivo*. Microbiome analyses show the interactions between complex microbial communities and our bodies. *In vivo* imaging provides insights into the mechanisms underlying the disease and the efficacy of treatments. Finally, artificial intelligence and machine learning are implied to analyse large and complex datasets generated by various omics technologies and to identify patterns and relationships that would be difficult or impossible to detect using traditional methods.

This mini-review aims to highlight the hot areas where novel technologies in biomedical research can have the most significant impact on AT research, thus enriching our understanding of AT function and improving the treatment of metabolic pathologies.

## 2. Single-cell sequencing

Single-cell sequencing (sc-seq) is a revolutionary tool with the potential to advance our fundamental understanding of how biological systems operate, particularly in AT research. Considering the diversity of AT and its heterogeneity, sc-seq techniques allow a unique view at a single-cell level to identify new cell types and gene expression patterns that may be involved in AT function and dysfunction.

sc-seq comprises a variety of techniques, including single-cell RNA sequencing (scRNA-seq), single-cell DNA sequencing (scDNA-seq), single-cell chromatin accessibility profiling (scATAC-seq), and single-cell proteomics (scProteomics). In addition, more precise single-cell sub-technologies such as single-nuclei RNA sequencing (snRNA-seq) or sc-DNA methylome sequencing provide a possibility for a further expansion of our research capabilities [1–3].

Recent advances in single-cell proteomics have expanded the scope of this field beyond cytometry [4,5]. The development of bottom-up proteomics [6,7] has allowed for a more detailed and comprehensive analysis of protein expression at the single-cell level. This technique involves the digestion of proteins into peptides, which are then identified and quantified using mass spectrometry. This approach provides a more in-depth understanding of the proteome, enabling researchers to identify novel protein interactions and pathways that may be involved in disease pathogenesis.

Among these technologies, scRNA-seq is most commonly used in AT research to identify and characterize various cell types, novel subpopulations, specific cell activation, and cellular responses to intervention. A good example of the promise of sc-seq techniques is the identification of novel therapeutic targets for cancer-associated lymphoedema by Liu *et al*. [8], which opens the door to the development of more targeted therapies for treating lymphoedema. Single-cell technologies, such as scRNA-seq and mass cytometry, allow the identification of specific subpopulations of mesenchymal stem cells (MSCs) and other cell populations within AT, such as immune and endothelial cells, which play important roles in tissue homoeostasis and disease pathogenesis. Gu *et al*. [9] identified two subpopulations of adipose-derived mesenchymal stem cells (ASCs) within perivascular AT (PVAT) using scRNA-seq. Since the PVAT phenotype is associated with atherosclerosis and other vascular disease progressions [10] and ASCs could be an important target for treating cardiovascular diseases [11], findings such as this may provide clues for novel therapeutic strategies for a variety of diseases. The importance of using single-cell technologies as a tool for analysing cell populations within the AT was highlighted in the study by Brooks *et al*. [12], who demonstrated that ASCs are a very heterogeneous group of cells, more diverse than previously appreciated.

Overall, the use of single-cell technologies has the potential to revolutionize our understanding of the heterogeneity of AT and its biology and improve our ability to develop targeted therapies for various diseases by identifying specific molecular targets [13].

## 3. Multi-omics analyses

Multi-omics analyses (including genomics, transcriptomics, proteomics, and metabolomics) allow for integrating multiple types of high-throughput biological data to comprehensively understand biological systems. Multi-omics analyses in AT research provide a unique opportunity to investigate the complex molecular interactions and cellular heterogeneity of AT related to energy homoeostasis, thus improving current therapeutic options. Multi-omics analyses can be pivotal in many areas of adipose research, such as investigating tissue heterogeneity, the molecular mechanisms of adipogenesis, AT inflammation and insulin resistance, thermogenesis and energy expenditure, and AT remodelling and plasticity.

The choice of omics layers for integration can significantly impact the knowledge gained from these analyses [14,15]. For instance, integrating genomics and transcriptomics can provide valuable information about how genetic variations influence gene expression patterns, which can be crucial for understanding disease mechanisms. On the other hand, combining proteomics and metabolomics can shed light on the functional consequences of gene expression changes, revealing the metabolic pathways that are affected in disease states.

However, it is important to note that not all combinations of omics layers provide unique information. For example, genomics and transcriptomics often show a high degree of correlation because changes in gene expression are frequently driven by genetic variations. Similarly, proteomics and metabolomics can also show redundancy, as changes in protein levels often lead to corresponding changes in metabolite levels. Therefore, researchers should carefully consider the specific research question and potential redundancy of information when choosing which omics layers to integrate.

In their study, Sundekilde *et al*. [16] used multi-omics integrated analysis of liver, muscle, AT, urine, plasma, and faeces samples from high-fat diet (HFD)-fed mice and defined key molecular pathway alterations in genes involved in lipid and energy metabolism and inflammation. Using multi-omics analysis, Yang *et al*. [17] found that distinct subpopulations of ASCs in separate AT depots obtained from Sprague Dawley rats exhibit different associations with fat metabolism and insulin resistance, reflecting differential contributions to these metabolic processes. Studies, such as those by Flenkenthaler *et al*. [18], demonstrate the power of multi-omics analyses in providing a comprehensive understanding of AT function and dysfunction.

Multi-omics analyses have helped to understand the clinical relevance of visceral AT in advancing colorectal tumour growth [19] or the effect of air pollution exposure on obesity [20]. The datasets created from multi-omics technologies serve as a valuable tool for comparative and translational research on metabolic diseases such as metabolic syndrome and type 2 diabetes mellitus (T2DM). Gene expression multi-omics data revealed the differential expression of multiple genes, microRNAs, and gene methylation in subcutaneous AT (SAT) from female individuals during ageing, thus providing a landscape for further research on ageing and tissue repair [21].

Multi-omics technologies offer a unique opportunity to investigate the complex interplay between different biological pathways and provide holistic insights into AT function in health and disease. These technologies integrate data from genomics, transcriptomics, proteomics, metabolomics, and microbiomics. As genomics reveals gene variations that may predispose to certain diseases or affect the response to treatment, transcriptomics takes into account the complete set of RNA transcripts, giving information about the functional molecules that carry out most life processes. Multi-omics technologies also include advanced techniques to investigate post-translational modifications (PTMs) at a global level, including processes such as phosphorylation, acetylation, and ubiquitination, which play crucial roles in regulating protein function and activity. Additionally, by examining the entire set of expressed proteins, proteomics highlights specific functional molecules involved in specific processes. On the other hand, metabolomics reflects the end products of cellular processes by focusing on the complete set of small-molecule chemicals. Furthermore, microbiome analyses show the interactions between complex microbial communities in the body. By integrating multi-omics data with other types of information, such as clinical data and environmental factors, today, we can gain a more comprehensive understanding of the factors that influence AT function in health and disease.

Comprehensive biological approaches are essential for understanding metabolic conditions, such as obesity, metabolic syndrome, and T2DM at the organism level, which can ultimately lead to the development of more personalized approaches for treating metabolic disorders.

## 4. Spatial omics technologies

Spatial omics, including spatial transcriptomics, special proteomics, and spatial metabolomics, are highly useful tools for precisely determining tissue structure, aiming to clarify the distinct subpopulations and their metabolic differences within intact tissues [22,23].

Imaging-based spatial transcriptomics technologies involve *in situ* hybridization (ISH) of mRNAs to fluorescently labelled specific probes or *in situ* sequencing (ISS) of amplified mRNAs inside a tissue section by sequencing by ligation (SBL) technology of fluorophore-labelled 1–2 bases at a time. These methods include MERFISH (multiplexed error-robust fluorescence *in situ* hybridization), FISSEQ (fluorescent *in situ* sequencing), seqFISH+ (an advanced version of sequential fluorescence *in situ* hybridization), and ExSeq (expansion sequencing) [24–27]. Sequencing-based spatial transcriptomics methods directly detect the location by ligation of mRNAs to spatially barcoded probes, followed by reverse transcription of captured mRNA resulting in cDNA with a spatial barcode of the primer, next-generation sequencing (NGS), and localization of each mRNA transcript within the tissue section using the spatial barcode. Recent methods include Geo-seq (geospatial transcriptomics sequencing), HDST (high-definition spatial transcriptomics), and Slide-seqV2 (an improved version of Slide-seq) [28–30].

Using spatial transcriptomics on human white AT, Bäckdahl *et al*. [31] distinguished adipocytes divided into two co-localized subpopulations, AdipoLEP (marker gene LEP encoding leptin playing a role in remodelling the extracellular matrix) and AdipoPLIN (genes PLIN1/PLIN4 for proteins perilipins 1 and 4 involved in lipid and glucose metabolism, insulin response, and the insulin-sensitizing hormone adiponectin), as well as a separate subpopulation, AdipoSAA (involved in the pro-inflammatory effects). It was determined that the obese, compared to non-obese subjects, had fewer AdipoPLIN cells, confirming that the proportion of subpopulations is affected by obesity [31]. Langin [32] suggests that, since the gene expression in the method is visualized as coloured dots, smaller dots and hence a better resolution can yield a refined spatial mapping and even clarify the transition between types, indicating the possibility of determining whether AdipoPLIN is converted to AdipoLEP adipocytes that are metabolically unfit.

The main limitation of spatial transcriptomics is that the assays are not based on a single-cell level; therefore, the transcripts at certain positions may originate from heterogeneous cells. To determine adipocyte subpopulations, spatial transcriptomics should be combined with different methods, such as sc-seq or snRNA-seq [33]. Andersson *et al*. [34] provided an open-source Python package, stereoscope, that integrates scSeq and spatial transcriptomics data, deconvolution of mixed expression profiles, and spatial mapping of cell types.

Spatial proteomics is an emerging field that aims to investigate the spatial distribution of proteins within cells and tissues. This approach can provide information regarding protein localization and function, which is crucial for understanding cellular processes and disease mechanisms. One spatial proteomics method is based on the use of engineered peroxidases, such as ascorbate peroxidase (APEX), which can be genetically targeted to specific cellular compartments of interest [35]. Combined with ratiometric tagging strategies, this method allows the mapping of proteomes with nanometre spatial resolution. The field of spatial proteomics has also benefited from machine-learning techniques. Machine learning algorithms have been used to analyse spatial proteomic data and improve our understanding of protein localization and dynamics [36].

On the other hand, spatial metabolomics focuses on the spatial distribution of metabolites within biological systems. This technique can provide valuable information about metabolic pathways and their spatial organization within tissues, which can help understand disease mechanisms and identify potential therapeutic targets [37,38].

Together, spatial omics technologies can transform our understanding of AT function in health and disease, leading to more effective treatments for a wide range of conditions.

## 5. Live imaging

Live imaging techniques have revolutionized biomedical research, enabling scientists to understand complex biological systems by preserving the structural integrity of tissues, ensuring comprehensive and holistic investigation.

Nishimura *et al*. [39], for the first time, used a real-time imaging technique based on confocal laser microscopy to analyse the structures, cellular dynamics, and vascular function of lean and obese AT within living mice, concluding that visceral AT obesity is an inflammatory disease with altered microcirculation and cellular dynamics. In addition to analysing structures, cellular dynamics, and vascular function, this technique could potentially be harnessed to visualize PTMs in AT within living organisms, thereby providing real-time insights into protein regulation in the context of obesity and other metabolic diseases. Brooks *et al*. [40] investigated the kinetics of adipogenesis using a live-cell imaging system of human ASCs combined with a deep learning-based detection tool. During adipogenesis, images were obtained by a deep learning system to annotate regions of interest using algorithms that detect the adipose area, total cell area, and lipid droplets, which were later compared with the morphological changes and genetic expression of ASCs at different time points during differentiation. Lipid droplets during adipocyte differentiation in pathophysiological conditions, such as obesity, T2DM, atherosclerosis, fatty liver diseases, and heart disease, can be analysed by label-free stimulated Raman scattering microscopy and three-dimensional sectioning [41]. Deep learning-based image analysis in live-cell imaging allows quantification of adipogenic differentiation kinetics and can also potentially detect and analyse PTMs in adipose-derived stem/stromal cells [42,43]. Understanding these modifications provides further information into the regulation of proteins involved in adipogenesis and metabolic disturbances.

Together, live-cell imaging approaches can transform our understanding of disease processes and lead to more effective treatments for a wide range of conditions. These methods contribute to a better understanding of the kinetics of processes that lead to metabolic disturbances, tissue dysfunction, or the impact of pharmacological interventions.

## 6. 3D tissue engineering

3D cell culture is a bioengineering approach to creating functional tissues using living cells and biomaterials. Although technically more challenging, it has many advantages over two-dimensional cell culture, such as unaltered cellular morphology, well-characterized differentiation, improved cell viability and response to stimuli, and native-like drug metabolism, gene expression, protein synthesis, and cellular proliferation [44,45]. Matrix- and scaffold-based or scaffold-free constructs made from adipose-derived cells, microfluidic devices that simulate interstitial flow and shear stress in AT, and fat-on-a-chip systems with multiple cell types and organoids are some examples of AT platforms created via 3D stromal or stem cell culture techniques [46].

3D AT models with *in vivo* relevance enable the investigation of adipogenesis, adipocyte metabolism, obesity, and obesity-related diseases [47]. For instance, a scaffold-free spheroid 3D culture from primary human or murine preadipocytes closely matches *in vivo* adipogenesis [48]. Moreover, cultures of adipocytes and other cell types could provide insights into the metabolic relationship between obesity, T2DM, and inflammation. A 3D co-culture system of adipocytes and macrophages was characterized by insulin resistance [49]. Quantitative proteomic data from this *in vitro* obesity model revealed that it could be utilized to investigate the pathophysiological mechanisms associated with obesity and perform drug screening. Bio-printed and other types of 3D AT models could be used to study the crosstalk between adipocytes and cancer cells and how this affects tumour growth, invasion, and metastasis, as well as some cancer-associated pathologies [47,50,51]. Recent advancements in organ-on-a-chip technology have enabled the construction of microphysiological systems, which hold promise for substituting animal models for high-throughput drug testing for human diseases. Systems with the functional properties of a human-like adipose depot have been validated [52,53]. Similarly, human adipose-derived stem cells successfully differentiated ‘on-chip’, and the integrated microfluidics enabled interstitial flow support of the 3D microtissue, along with the options to simulate changes in the internal environment, *e.g*., blood glucose oscillations, and non-destructively acquire samples for proteomic analysis [54,55]. 3D AT models can also serve as an excellent platform for studying post-translational modifications in a more physiologically relevant context [56,57]. Such models may enhance our understanding of how PTMs regulate adipocyte metabolism, obesity, and obesity-related diseases.

Overall, the utilization of functional AT models for drug testing or as a platform for investigating AT development and disease is possible using 3D tissue engineering techniques. These methods can produce constructs of AT that are physiologically relevant and closely resemble the native microenvironment and interactions between adipocytes and other cell types.

## 7. Microbiome analysis

The microbiome has emerged as a critical player in the regulation of various physiological processes, and growing evidence supports its role in AT function and metabolism. Recent advancements in high-throughput sequencing and bioinformatics have paved the way for the comprehensive analysis of complex microbial communities inhabiting the human body.

The gut microbiota plays an essential role in modulating AT. The imbalance of microbes due to alterations in diet and environmental factors results in dysbiosis, which disrupts intestinal barrier function, causes chronic inflammation, and results in metabolic disorders such as obesity. Obese individuals have a lower abundance of Bacteroidetes and a higher proportion of Firmicutes than non-obese subjects [58]. Germ-free (GF) mice show 60% greater body fat mass compared to conventional mice [59], and gut microbiota transplants from obese to GF mice led to greater fat mass and body weight compared to lean donors [60]. HFD in mice causes dysbiosis and intestinal barrier disruption, leading to higher levels of bacterial endotoxins and lipopolysaccharides (LPSs) [61]. Higher levels of LPS from the HFD led to elevated levels of F4/80-positive cells and inflammatory markers in white AT (WAT) [62]. LPSs activate toll-like receptor 4 (TLR4) in adipocytes, increasing interleukin 6 (IL-6) and tumour-necrosis factor alpha (TNFα) levels and activating proinflammatory pathways, such as NF-κB signalling, leading to AT inflammation. Metabolites from the gut microbiota, such as short-chain fatty acids (SCFAs), polyamines, and aryl hydrocarbon receptor (AHR) ligands, can decrease LPS-associated inflammation [63].

The gut microbiota also affects adipokine secretion. Microbiota transplantation in GF mice resulted in higher fat mass and proportionally higher leptin levels [64]. Narmaki *et al*. [65] showed decreased leptin levels compared with baseline values after 12 weeks of probiotic treatment in obese women. Yao *et al*. [66] found that altering the gut microbiota with antibiotics resulted in higher mRNA levels of adiponectin, inhibition of weight gain, and greater expression of thermogenic and lipid oxidation genes such as peroxisome proliferator-activated receptor alpha (PPARα) in ATs.

The rapidly evolving field of microbiome research holds significant potential to advance our understanding of AT biology and its role in health and disease. The interaction between the gut microbiota and AT is becoming increasingly apparent and can be used as a potential target for therapeutic interventions and the treatment of obesity and related metabolic disorders. Recent advancements in metaproteomics and metabolomics have further enriched this field, allowing for a more comprehensive understanding of the functional dynamics of the microbiome [67,68]. Metaproteomics gives information on the protein expression profiles of the microbiota, whereas metabolomics allows for the study of metabolites produced by these microorganisms. Combined with traditional genomic approaches, these techniques offer a holistic view of the microbiome and its interactions with the host, thus opening new prospects for therapeutic interventions [69,70].

## 8. *In vivo* imaging

*In vivo* imaging refers to the non-invasive monitoring of AT dynamics in living organisms, which is useful for developing novel diagnostic and therapeutic strategies.

The most commonly used *in vivo* imaging systems, magnetic resonance imaging (MRI) and computed tomography (CT), are associated with the high cost of equipment, radioactivity, low sensitivity in some cases, and inability to be used in studies where repeated scans are required. Therefore, new methods and dyes are required for this purpose.

Specifically, CT and MRI have been recognized as the gold standards for evaluating body composition because of their ability to separately quantify different compartments of AT, such as visceral AT (VAT) and SAT. These methods provide a detailed view of the distribution and volume of AT, allowing for precise quantification and characterization [71]. CT can differentiate between various types of tissues based on their density. This method is especially effective for quantifying VAT, which is closely associated with metabolic disorders and cardiovascular diseases. In contrast, MRI offers superior soft tissue contrast without exposure to ionizing radiation, making it ideal for longitudinal studies. This method can accurately measure total body fat, SAT, and VAT [72].

However, these imaging techniques are expensive and require sophisticated equipment. To overcome these limitations, new methods and dyes have been developed. For instance, Jo *et al*. [73] proposed a method for near-infrared (NIR) fluorescence imaging of brown AT (BAT) in living animals using a new sensitive and non-radioactive dye, BF800-AM. In another study, multispectral optoacoustic imaging technology with ultrasound tomography (MSOT-US) detected an expressed optoacoustic contrast agent, a near-infrared fluorescent protein (iRFP). iRFP has been used for the *in vivo* monitoring of dynamic changes in lipid metabolism triggered by browning activation in white adipocytes during adrenergic stimulation [74].

The anatomical, morphological, and functional aspects of AT in animals and humans can be studied using *in vivo* imaging techniques [75]. Different techniques can be combined to obtain complementary information and overcome individual limitations.

Elastography ultrasound can be used to measure SAT fibrosis, demonstrating the pathological changes linked to the development of obesity [76]. Computed tomography (CT), dual-energy X-ray absorptiometry (DEXA), and especially MRI can be used to determine AT volume, distribution, or fatty acid composition. Examples include their use in studies on the effects of dietary and pharmacological interventions on obesity [77,78], non-alcoholic fatty liver disease, non-alcoholic steatohepatitis [79], and T2DM [80]. MRI can be complemented by *in vivo* magnetic resonance spectroscopy to provide further metabolic insights [78,81].

Dual-energy CT, MRI, positron emission tomography (PET), optoacoustic imaging, near-infrared spectroscopy, contrast-enhanced ultrasound, Cerenkov luminescence imaging, fluorescence imaging, and combinations thereof can discriminate between WAT and BAT [75,82–84]. For example, an optoacoustic approach has been used to study the effects of BAT in T2DM [85]. Specific injected positron-emitting radiotracers can enter various metabolic pathways, enabling BAT determination and investigation of their role in metabolism. PET can be used to monitor glucose utilization, fatty acid oxidation, adipokine secretion, inflammation, perfusion, and angiogenesis in AT. For instance, it has been successfully used to investigate dietary intake and BAT activity [86], the role of BAT in cancer activity [87], and the interaction of thyroid hormones with BAT [88].

*In vivo* imaging methods for AT entail access to costly, sophisticated equipment, produce large amounts of data requiring the creation and optimization of data processing algorithms, often including the use of AI, and may involve exposure to ionizing radiation. However, they are valuable tools that provide spatial and temporal information for understanding AT function in health and disease.

## 9. Artificial intelligence

Artificial intelligence (AI) is a broad term that refers to the simulation of human intelligence processed by machines, particularly computer systems. Machine learning (ML) is a subset of AI that involves the use of statistical techniques to enable machines to improve with experience. Deep learning, a further subset of machine learning, inspired by the brain’s natural networks and uses artificial neural networks (ANNs), thus adding additional layers to optimize the accuracy of MR [89,90].

AI with ML algorithms can analyse large-scale datasets, including imaging data, to identify and quantify the different compartments of AT. AI can be used to differentiate between WAT and BAT, as well as to identify and quantify other components of AT, such as immune cells, blood vessels, and extracellular matrix [89]. ML algorithms can be trained to analyse imaging data from CT, MRI, or positron emission tomography/computed tomography (PET/CT) analyses to accurately quantify the volume and distribution of different AT compartments.

AI and ML are powerful tools for analysing datasets, such as those generated by various omics technologies (transcriptomics, proteomics, *etc*.).

ML algorithms can identify subtle changes in gene expression, metabolite levels, or protein structures associated with disease, even if those changes are not statistically significant on their own [91]. ML has potential application in identifying new biomarkers for metabolic diseases and pinpointing specific patterns in gene expression, metabolite levels, or protein structures that are unique to certain types of metabolic diseases. This could allow for more accurate diagnosis and treatment [92].

Furthermore, ML can help identify new therapeutic targets [91,92]. For example, machine learning algorithms could be used to identify molecular pathways that are disrupted in metabolic diseases and then target those pathways with drugs to restore normal function [92–94]. Using AI and machine learning, patterns and correlations in PTM data can be identified, thus gaining essential information about the molecular mechanisms underlying metabolic diseases associated with AT dysfunction [95,96].

However, there are several challenges associated with using ML to analyse large-scale datasets, including the need for high-quality data, the difficulty of interpreting complex models, and the potential for bias or overfitting. In addition, there are ethical and regulatory issues associated with the use of ML algorithms in healthcare, particularly for data privacy and security [97]. Despite these challenges, there has been significant progress in recent years in using AI to analyse transcriptomics and metabolomics data to gain new insights into metabolic diseases associated with AT dysfunction.

As the field continues to evolve, AI will likely play an increasingly important role in identifying new biomarkers and therapeutic targets for metabolic diseases, ultimately improving patient outcomes.

## 10. Conclusions

Novel technologies based on advances in molecular biology, nanotechnology, and informatics are great tools for expanding our knowledge of the mechanisms by which AT functions. Combining these methods allows us to better understand the mechanisms and regulatory networks governing AT development and function. By integrating scRNA-seq with other omics data, such as chromatin accessibility, DNA methylation, or protein expression, it is possible to infer the transcriptional and epigenetic factors that control cell fate decisions and functional phenotypes in AT. Moreover, by applying computational methods to infer cell-cell interactions from scRNA-seq data, it is possible to reconstruct cellular crosstalk and communication within the AT microenvironment. For example, scRNA-seq revealed that adipocytes secrete various factors that can modulate the polarization and activation of macrophages in AT. Conversely, macrophages can produce cytokines and chemokines that affect adipocyte differentiation and metabolism. Understanding these bidirectional interactions may help identify novel therapeutic targets for metabolic diseases.

Despite the advantages offered by these novel technologies, they face several challenges and limitations that need to be addressed in future studies. Currently, the data obtained may still be noisy, sparse, often incomplete, and sometimes difficult to interpret. Therefore, comprehensive sampling strategies, experimental designs, appropriate statistical methods, and biological validation are required to capture the full diversity and complexity of AT.

## List of abbreviations

3D: 3-dimentional
AI: Artificial intelligence
AHR: Aryl hydrocarbon receptor
ANN: Artificial neural networks
APEX: Ascorbate peroxidase
ASC: Adipose-derived mesenchymal stem cells
AT: Adipose tissue
BAT: Brown adipose tissue
CT: Computed tomography
DEXA: Dual-energy X-ray absorptiometry
ExSeq: Expansion sequencing
FISSEQ: Fluorescent *in situ* sequencing
Geo-seq: Geospatial transcriptomics sequencing
GF: Germ-free
HFD: High-fat diet
HDST: High-definition spatial transcriptomics
IL-6: Interleukin 6
iRFP: Near-infrared fluorescent protein
ISH: *In situ* hybridization
ISS: *In situ* sequencing
LPS: Lipopolysaccharides
MERFISH: Multiplexed error-robust fluorescence in situ hybridization
ML: Machine learning
MRI: Magnetic resonance imaging
MSC: Mesenchymal stem cell
MSOT-US: Multispectral optoacoustic imaging technology with ultrasound tomography
NGS: Next-generation sequencing
NIR: Near-infrared (fluorescence imaging)
NF-κB: Nuclear factor kappa-light-chain-enhancer of activated B cells
PET: Positron emission tomography
PET/CT: Positron emission tomography/computed tomography
PPARα: Peroxisome proliferator-activated receptor alpha
PTM: Post-translational modification
PVAT: Perivascular adipose tissue
SAT: Subcutaneous adipose tissue
SBL: Sequencing by ligation
scATAC-seq: Single-cell chromatin accessibility profiling
scDNA-seq: Single-cell DNA sequencing
SCFA: Short-chain fatty acid
scProteomics: Single-cell proteomics
scRNA-seq: Single-cell RNA sequencing
sc-seq: Single-cell sequencing
seqFISH+: An advanced version of sequential fluorescence *in situ* hybridization
Slide-seqV2: An improved version of Slide-seq
snRNA-seq: Single-nuclei RNA sequencing
T2DM: Type 2 diabetes mellitus
TLR4: Toll-like receptor 4
TNFα: Tumor necrosis factor alpha
VAT: Visceral adipose tissue
WAT: White adipose tissue
